# Linking Extracellular Matrix Agrin to the Hippo Pathway in Liver Cancer and Beyond

**DOI:** 10.3390/cancers10020045

**Published:** 2018-02-06

**Authors:** Sayan Chakraborty, Wanjin Hong

**Affiliations:** Institute of Molecular and Cell Biology, Agency for Science, Technology and Research (A-STAR), 61 Biopolis Drive, Proteos, Singapore 138673, Singapore; mcbhwj@imcb.a-star.edu.sg

**Keywords:** Hippo pathway, agrin, YAP/TAZ, mechanotransduction, liver cancer, extracellular matrix, cardiac regeneration, neuromuscular junctions

## Abstract

In addition to the structural and scaffolding role, the extracellular matrix (ECM) is emerging as a hub for biomechanical signal transduction that is frequently relayed to intracellular sensors to regulate diverse cellular processes. At a macroscopic scale, matrix rigidity confers long-ranging effects contributing towards tissue fibrosis and cancer. The transcriptional co-activators YAP/TAZ, better known as the converging effectors of the Hippo pathway, are widely recognized for their new role as nuclear mechanosensors during organ homeostasis and cancer. Still, how YAP/TAZ senses these “stiffness cues” from the ECM remains enigmatic. Here, we highlight the recent perspectives on the role of agrin in mechanosignaling from the ECM via antagonizing the Hippo pathway to activate YAP/TAZ in the contexts of cancer, neuromuscular junctions, and cardiac regeneration.

## 1. Introduction

The extracellular matrix (ECM), defined as the non-cellular component of the tissue microenvironment, is an important contributor to the overall microenvironment. Owing to its diverse biochemical and mechanical properties, the ECM and its soluble component(s) dictate cell behavior that is important in cancer and organ development [1,2]. The mechanoreception of cell-cell and cell-ECM interface controls cellular shape, organization and proliferation [3,4,5]. Within normal epithelial organs, tissue architecture has been proposed to act as an inherent tumor-suppressor, thereby confining and tightly regulating mechanical constraints [6]. In contrast, a growing tumor deviates from normal tissue architecture rendering it to be more inflamed, compressed, and rigid [7]. Despite an altered mechanoresponse widely demonstrated by most cancer cells and within tumor tissues, the identities of soluble ECM proteins that mediate mechanosignalling during cancer progression remain elusive. 

The transcription factors YAP and TAZ (encoded by *YAP1* and *WWTR1* genes, respectively) that are normally restricted by the conserved Hippo tumor-suppressor pathway, are activated in diverse cancers and are increasingly associated with nuclear sensors for biomechanical signals [8,9,10,11]. In general, the role of YAP/TAZ is well understood as converging effectors of the Hippo pathway. The mammalian Hippo pathway core machinery consists of Serine/Threonine kinases Mst1/2 (also referred as STK4) and Large tumor suppressor 1 and 2 (LATS1 and LATS2), together with their respective adaptor proteins Salvador (SAV1/WW45) and MOB (MOB1A/1B) serving as tumor suppressor proteins [12,13,14]. In response to multiple upstream signals, activated LATS1/2 phosphorylates YAP/TAZ at the “HXRXXS” motifs, thereby sequestering them in the cytoplasm and/or priming for ubiquitination-mediated proteosomal degradation [13,15,16,17,18]. YAP/TAZ binds to transcriptional enhancer factor (TEF with TEA domain), commonly referred as TEAD transcription factors to activate a transcription program comprising of hundreds of genes that are generally involved in promoting cell proliferation and inhibiting apoptosis [19,20,21,22,23,24,25]. Owing to their pro-survival functions, YAP/TAZ is widely implicated in most solid cancer types where they regulate tumor initiation, progression and metastasis, and stemness [26]. Over a decade of active research has elucidated the key upstream effectors and spatial distribution of the Hippo pathway that strike an intracellular balance, thereby governing YAP activity in different contexts [12,27]. Hence, a myriad of oncogenic signaling pathways including Wnt, G-protein coupled receptors (GPCRs), and epidermal growth factor (EGF) hijack the Hippo tumor-suppressor pathway to positively regulate YAP/TAZ to promote tumorigenesis and progression [28,29,30,31]. Intriguingly, YAP/TAZ are at the cross-roads of multiple inputs and hence sense cues that include ECM stiffness and remodeling, cell geometrical constraints, and cytoskeletal induced mechanical changes that may be dependent or independent of the Hippo pathway [9,10,30,32,33,34,35]. The capabilities of YAP and TAZ in reading a wide array of mechanical inputs and converting them into defined biological responses are enormous. However, the real challenge lies in the identification of key ECM factors(s) serving as upstream inputs and the precise mechanosignalling pathway to nuclear YAP/TAZ.

**Figure 1:**
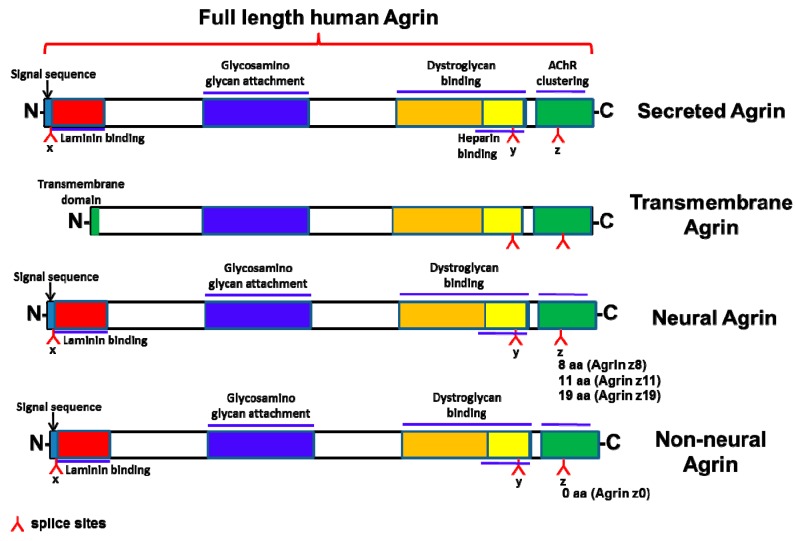
Cartoon showing the different forms of Agrin.

The heparan sulfate proteoglycan, agrin, is best known for its ability to cluster acetylcholine (AChR) receptors in the neuromuscular junctions (NMJ). Agrin binds to Lipoprotein-related receptor-4 (Lrp4) to activate Muscle-specific tyrosine kinase (MuSK), thereby forming a multiple-protein complex within the NMJs [36,37,38]. As such, agrin is essential for NMJ formation early during developmental stages in vivo [39]. It binds to the N-terminus of Lrp4 to stimulate the auto-phosphorylation of its co-receptor MuSK, critical for the maintenance of NMJs [40]. Agrin exists as a secreted protein which encodes a signal peptide followed by the N-terminus, or as a shorter Type II Transmembrane (Tm) form with an internal signal peptide (38) (Figure 1). Alternative splicing in at-least two positions within the C-terminus of Agrin known as “y” and “z” sites, respectively, enables dystroglycan, Lrp4-MuSK and possibly integrin receptor(s) engagement (Figure 1) [38,41,42]. However, the most critical and well-defined isoforms are the ones bearing insertions of 8, 11 and 19 (8+11) amino acids at the “z” site that potently activate MuSK through Lrp4 recruitment [38]. These isoforms are broadly referred to as “neuronal” Agrin. Binding of neuronal Agrin to Lrp4 involves a synergistic formation of a tetrameric complex mediated by “z8 loop” [43]. Muscle variants that lack “z” inserts are far less robust in binding Lrp4 and activating MuSK [42]. On the other-hand, the Lys-Ser-Arg-Lys-residues at the “y” site are critical for heparin and α-dystroglycan engagement (38). Irrespective of isoform distinctions, both muscle and neuronal Agrin are known to bind laminin and promote cytoskeletal rearrangements with mechanisms that remained largely unknown [44,45]. 

Despite its widely recognized function in the establishment and maintenance of NMJs, the role of agrin outside the neuromuscular interface is less clear. In addition to the accumulation of agrin in the liver of rats undergoing chemically induced cirrhosis and hepatocellular carcinoma (HCC) [46], recent advancements in the field discovered a non-canonical function of agrin as an extracellular matrix sensor that stabilized focal adhesions and promoted HCC [47]. Surprisingly, agrin also hijacks its neuronal receptor machinery (Lrp4/MuSK) and combines with the integrin-Focal adhesion kinase (FAK) mechanosensing complex to form an oncogenic axis in liver cancer; therefore, explaining its frequent overexpression and secretion observed amongst liver cancer patients. Importantly, agrin depletion reduced in vivo liver tumor growth, suggesting its role in HCC progression [47]. In this perspective, YAP is amplified in several cancers including HCC [48]. Chronic expression of YAP and its phosphorylation defective mutant enlarges liver size that culminates in HCC development by perturbing the Hippo pathway [8,49,50]. As a key mechanosensing complex, the integrin-FAK signaling is also implicated in the activation of YAP [51,52,53]. Therefore, the combination of these seminal findings raised a possibility of a mechanotransduction network between agrin and YAP that underscores the oncogenic properties of liver cancer. 

The role of proteoglycans in activating YAP/TAZ or mediating cell/matrix stiffness has not been investigated thus far. However, recent shreds of evidence serve the basis for a greater understanding on the role of agrin as a secreted proteoglycan on YAP functions in liver cancer and other tissues. Agrin as an ECM signal is recognized by the integrin/Lrp4/MuSK receptors, which is necessary and sufficient to sustain the activity of YAP in response to mechanical changes [54,55]. During this process, agrin inhibited the Hippo tumor suppressor pathway leading to enhanced activity of YAP. Mechanistically, these findings demonstrate that agrin requires YAP-mediated transcriptional activity for its oncogenic property, making it clinically relevant for liver cancer progression. Here, we discuss the implications of the newly discovered role of agrin and YAP activity in the context of liver cancer, NMJs, and cardiac regeneration.

## 2. Newly Discovered Role of Agrin and YAP

### 2.1. Agrin Activates and Stabilizes YAP

How ECM signals regulate the activity of nuclear YAP/TAZ are interesting to understand the relay of mechanosignalling from ‘outside the cell’ to intracellular YAP/TAZ. The role of agrin in the regulation of any transcriptional co-activator has not been previously reported. In fact, not many proteoglycans have been reported so far that directly affect YAP/TAZ activity. Hence, the emerging studies on agrin reveal interesting mechanistic insight onto this aspect. For instance, agrin depletion was suggested to promote an inhibitory phosphorylation of YAP at Serine127 residue that shifted nuclear YAP into the cytoplasm, a key step in the regulation of YAP activity by the Hippo pathway [18]. Additionally, function-blocking antibodies that prevent agrin’s interaction with its receptor repertoire similarly inhibited YAP activity in vitro and in vivo and reduced tumor cell growth [54]. Interestingly, the YAP inactivation was highly correlated with decreased target gene expression in agrin knockdown cells, and the phenotype was significantly rescued by supplementing soluble neural or non-neural agrin (that are distinguished by the presence or absence of an eight amino acid insert within the Z exons). It is interesting that both neural and non-neural isoforms of agrin activate YAP, suggesting the broader impact of agrin on YAP in other tissues as well. 

Cell adhesions represent regions of high compression and tensile strength. Several junctional proteins including the Angiomotin (AMOT) family members regulate YAP in conjunction with actin-cytoskeletal changes. AMOT proteins can directly bind to YAP and negate its activity [56,57]. Binding of AMOT with F-actin competes with YAP:AMOT complex, thereby releasing YAP from the inhibitory association of AMOT and progressing into the nucleus [58,59,60]. Interestingly, LATS1/2-mediated phosphorylation of AMOT and disruption of actin-fibers antagonizes the AMOT: F-Actin binding [58,59]. In addition, AMOTs also activate LATS2 to sequester YAP to tight junctions [61]. Consistent with these findings, agrin depletion enhanced the YAP:AMOT association in the cytoplasm, and subsequently led to YAP inactivation [54]. These findings suggest that agrin antagonizes the YAP: AMOT interaction to maintain functionality of YAP. In addition to Hippo pathway mediated phosphorylation of YAP/TAZ facilitating binding of 14-3-3 proteins and cytoplasmic sequestration [62,63], phosphorylation of YAP at Serine 381 primes it for phosphodegron mediated phosphorylation, ubiquitination and proteosomal degradation [18]. Subsequently, LATS phosphorylation also primes YAP/TAZ for CK1σ/ε and β-TrCP mediated degradation [18,64]. Accordingly, silencing agrin in the liver cancer cells enhanced YAP Ser381 phosphorylation and de-stabilization of YAP; suggesting that cytoplasmic YAP sequestered by angiomotin and 14-3-3 complexes is likely subjected to proteasomal degradation in response to agrin depletion. The YAP cytoplasmic sequestration and degradation antagonized by agrin account likely for increased YAP stability in cancer cells having agrin overexpression. Whether agrin negatively regulates other junctional proteins involved in the Hippo pathway to release YAP from their inhibitory associations will be an important aspect of future analysis.

### 2.2. Mechanisms of Agrin Regulation on YAP Functions

#### Mechanoactivation of YAP in Response to Agrin from the ECM

**Figure 2:**
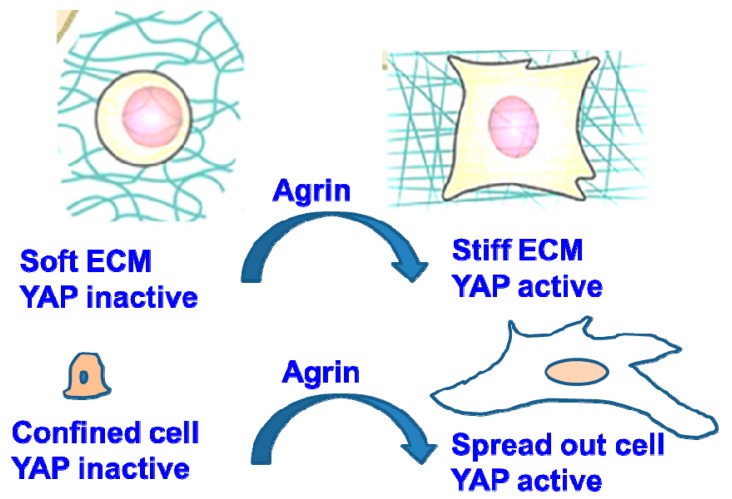
Role of agrin in maintaining YAP localization under altered Extracellular matrix ECM and cell geometrical shapes.

**Figure 3:**
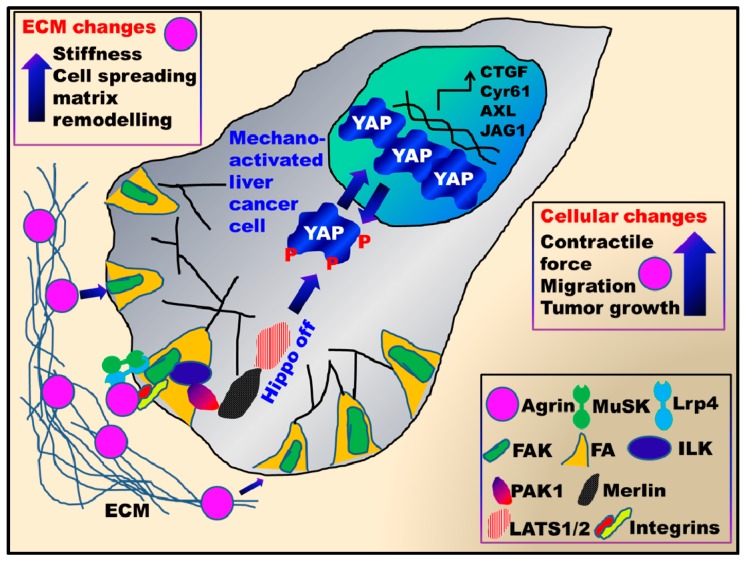
Coordinated mechanotransduction network involving agrin and YAP in liver cancer. Elevated agrin levels enhance ECM stiffness and remodeling in the liver by activating YAP. Soluble agrin binds to Lrp4/MuSK and integrins in liver cancer cells and stabilizes focal adhesions by activating Focal adhesion kinase-Integrin linked kinase- p21-Activated kinase (FAK-ILK-PAK1) axis. This activated mechanosignalling pathway inhibits the core Hippo components Merlin and LATS1/2 kinases. Further, agrin mediated mechanosignalling enhances cellular contractility and confer matrix stiffness by “mechano-activating” YAP mediated transcription. Together, agrin and YAP mediate changes in the cellular microenvironment that cumulatively enhance proliferation, migration and liver tumorigenesis.

A growing body of evidence indicates that ECM stiffness, cell spreading and cytoskeletal tension activate YAP by sustaining its nuclear localization and transcription of its target gene(s) [9,10,65]. Moreover, as compliant normal liver tissue matrices do not possess a well-defined basement membrane, excessive accumulation of agrin in addition to fibrillary collagen, laminin and perlecan will lead to the formation of a stiffer ECM during liver cancer development [66]. To understand how agrin and related proteoglycans stiffen up the liver ECM during cancer development, an existence of a functional mechanotransduction network between extracellular agrin and intracellular YAP was hypothesized. Importantly, the effects of subcellular YAP/TAZ localization are reflected in HCC spheroids cultured in three dimensions (3D). In such models, ECM stiffness (manipulated by enhancing collagen matrix concentration) increased the expression of agrin and its co-receptors in HCC cell lines that correlated with higher YAP activity [54]. Agrin depletion in cells cultured in stiff ECM reduced YAP’s nuclear localization and transcriptional activity and were consistently observed in stiffened 2D and 3D matrix. Conversely, supplementing agrin to cells cultured on either highly compliant matrices or geometrically confined areas was sufficient to activate YAP. Classically, cells that are stretched across a large surface area (10,000 μ^2^) harbor nuclear YAP and are characterized by enhanced YAP/TAZ signature gene(s) expression [9,67]. Depletion of agrin in liver cancer cells plated on large fibronectin islands shifted YAP into the cytoplasm and reduced the YAP-target gene expression [54]. Conversely, supplementation with soluble agrin potentiated nuclear YAP localization in cells that were confined to small fibronectin islands (<300 μ^2^). More importantly, agrin also stiffened the local ECM and provided considerable contractile strength to the cancer cells (Figure 2 and Figure 3). How cell geometry alters YAP/TAZ localization still remains unclear. Physical constraints, differential force transmission affecting nuclear pores and shapes [68], and F-actin distributional changes may represent the underlined mechanisms. Hence, further studies to delineate how agrin functions to cope up YAP activity within the geometrically constraint cells and how agrin mediated pathways generate force that is sensed by YAP/TAZ or their regulators will shed more light. More importantly, though persistent YAP activation enlarges liver size, the response to YAP activation is not uniform in the liver [69]. Since agrin levels are very low in normal livers [46,47,54,70], it will be of interest to determine at which stage the liver cell types start to accumulate agrin to promote complex pathophysiological changes of the ECM that is involved in the development of HCC. 

As a secreted basement membrane component [38], agrin may collaborate with laminin, collagen XVIII, and perlecan to tether soluble growth factors such as vascular endothelial growth factors (VEGF), transforming growth factor-β and fibroblast growth factors, thereby serving as an important mechanosignalling depot [71]. Such a docking platform may be regarded as a sensing platform for intracellular YAP activity and oncogenic response as well. In this context, Platelet derived growth factor (PDGF) stimulated hepatic stellate cells have been shown to promote agrin- induced hepatocarcinogenesis [72]. Whether agrin-induced YAP activity is influenced by these stellate cells will be of potential interest. The specificity of agrin in regulating YAP mechanotransduction from this ‘stiffening platform’ is illustrated by the fact that both perlecan and fibronectin stimulation of integrins failed to rescue YAP inactivation when agrin was depleted in soft-substrates [54]. Whether agrin plays a role in disorganization of the basement membrane through YAP activation that is important for tumor progression and metastasis is yet to be investigated. Moreover, agrin is heavily glycosylated at multiple repetitive serine-glycine (Ser-Gly) residues, serving as glycosaminoglycan (GAG) attachment sites for heparan/chondroitin sulfate [73]. Whether glycosylation pattern of agrin serves a modulating role in YAP mechanotransduction, activity and oncogenesis would be interesting avenues to pursue in the future. 

Again, exploring the “dynamic” interactions between cancer cells and their ECM, remodeling of local ECM by altering collagen production is very frequently observed in cancer cells. Such ECM remodeling can be characteristically observed by gel-contraction assays in-vitro. Agrin conferred contractility to cancer cells that remodeled collagen lattices [54]. Agrin may thus contribute towards development of motile, invasive actin-rich protrusions known as invadopodia that guide invasiveness of liver cancer cell lines [47]. While invadopodia are known to be enhanced in rigid ECM [74,75], the underlined regulation of YAP transcription in this process remains unknown. Hence, how agrin-YAP activity contributes towards tumor invasiveness is an issue of outstanding interest that needs to be explored. 

### 2.3. Agrin and its Regulation on Integrin-Focal Adhesions

The focal adhesions (FA) containing integrin receptors are membrane domains facilitating cell: ECM interactions and transmission of mechanosignalling. Several studies have elucidated that focal adhesion kinase (FAK) activity may promote nuclear translocation of YAP/TAZ involving Rho-GEF and LATS1/2 kinase dependent pathways [52,76]. In line with this, agrin emerged as an interesting player integrating and sustaining mechanical signals through defined pathways to YAP/TAZ. Agrin sustained the mechanoresponsiveness of YAP by utilizing FAK phosphorylation in a stiffness sensed manner that correlated with three-dimensional (3D) cancer cell growth [54]. Moreover, agrin also provided contractile strength to cancer cells in a YAP dependent manner and conferred significant matrix stiffness to otherwise compliant collagen gels [54]. Therefore, it is not surprising to ascertain that agrin tightly maintains the integrity of FA in liver cancer cells which is critical for their underlying oncogenic property [47]. Essentially, cancer cells displayed fragmented focal adhesions and lack of FAK phosphorylation in response to agrin depletion, an effect that was restored by supplementation of soluble agrin in the matrix [47]. Indeed, activated FAK was also essential for YAP dependent cellular contractility mediated by agrin [54]. In vivo, agrin depletion also reduced the fibrillary collagen content, an indication that it activates YAP for stiffening and crosslinking the ECM during cancer development. However, the agrin induced stiffening may also be due to over-accumulation of collagen fibrils as downstream transcriptional targets of YAP/TAZ in fibrosis [77].

Further, integrin activation has been directly linked to YAP activity [78,79,80]. Hence, the role of integrins downstream of agrin in HCC progression would be an interesting mechanism. Indeed, a majority of agrin induced mechanoresponsive effects on YAP was abolished by silencing integrin β1 in HCC cell lines [54]. More importantly, pre-treatment of liver cancer cells with arginine-glycine-aspartate (RGD) peptides that block integrin activation also suppressed agrin induced nuclear localization of YAP [54]. More importantly, blocking integrin activation also inhibited agrin-YAP induced cancer cell contractility and collagen remodeling abilities. However, agrin partly utilized Lrp4-MuSK receptors when integrin signaling was compromised in such situations. Quite interestingly, depletion of Lrp4 and MuSK in HCC cell lines also resulted in enhanced YAP-Ser127 phosphorylation [54]. Similar to agrin, integrin α2β1 heterodimer is also overexpressed in HCC that inhibits the Hippo kinase Mst1 phosphorylation [81]. Together, these data support an existence of a coordinated mechanosignalling network between agrin, integrins and FAs that antagonizes the Hippo pathway to sustain the YAP activity. In addition to negating the Hippo pathway, this axis, in combination with the Lrp4-MuSK receptors, formed the major mechanotransduction pathway contributing towards YAP activation (Figure 3). Depletion of Lrp4 and MuSK in the HCC cell lines partially enhanced YAP phosphorylation, suggesting these may be novel regulators of YAP activity and the Hippo pathway. Intriguingly, the combined perturbation of integrin and Lrp4-MuSK completely inhibited YAP activity, and therefore, rendering these cells incompetent to cope with biomechanical changes. In addition, as soluble C-terminus fragment of agrin rescued YAP activity in agrin-depleted cancer cells further suggesting that agrin engages its receptors for mediating downstream effects on YAP. Hence, agrin collectively utilized integrin and Lrp4/MuSK pathways to relay mechanosignalling to YAP and serves as a converging point upstream of Lrp4-MuSK and integrin-FAK receptors.

These findings also raise several key questions that need further investigations in the broader context of Hippo-YAP signaling and agrin biology. Similar to collagens being transcriptional targets of YAP/TAZ, focal adhesion marker gene expressions and FA formation are also regulated by YAP [77,82]. Moreover, as YAP activity itself is known to promote ECM stiffness [65], the fact that supplementation of recombinant agrin exerts considerable stiffness to the local matrix surrounding liver cancer spheroids may be hypothesized as a result of active YAP/TAZ transcription [54]. Whether YAP activation sustains agrin expression to provide a positive feedback between YAP and agrin-triggered ECM stiffness is an exciting possibility. Agrin is highly secreted in the circulation of HCC patients [47], and whether YAP/TAZ through TEAD transcriptional factors enhances agrin expression and secretion in liver cancer will be interesting to examine. It may be speculated that YAP-TEAD binds to enhancer region, thereby increasing agrin expression and stiffening liver cancer tissues. However, further studies are needed to prove the existence of transcriptional regulation of YAP on agrin expression. Of note, the possibilities of other transcriptional factors working in conjunction with TEAD to control agrin expression cannot be excluded. Moreover, how critical is the agrin-YAP signaling towards altering mechanoresponse of cancer cells? Would this mechanotransduction network be targeted for liver cancer therapy? Is this agrin-YAP mechanotransduction network active in chemotherapy-resistant cancer cells, and targeting this loop may re-awaken cancer cell sensitivity to chemotherapy? Furthermore, whether agrin overexpression enlarges liver size by engaging YAP mediated transcriptional target(s) would also be an exciting future study. Hence, we are looking forward to an exciting avenue of research aimed at understanding the agrin-YAP pathway in the context of aggressiveness of cancers.

### 2.4. Mechanosignalling Scaffold Initiated by Agrin

Downstream to integrin receptors, the activation of Integrin-linked-kinase (ILK) has been shown to perturb the Hippo pathway. ILK stimulates phosphorylation of MYPT-PP1, and subsequently inactivates Merlin, an upstream component of the Hippo pathway that is conserved in both flies and mammals [83,84]. Merlin (also known as Neurofibromin 2 or NF2) is expressed across the nervous system and mutations of NF2 gene were originally associated with Neurofibromatosis type 2, that comprises a group of benign neural tumors [85]. Merlin is an Ezrin-Radixin-Moesin (ERM) family protein [86,87], that organize Hippo signaling at the plasma membrane [88]. Membrane recruitment of LATS1/2 by Merlin facilitates YAP phosphorylation that occurs irrespective of MST1/2 activation, and tumor suppressive activities are ensued [88]. The underlying mechanism of inactivation of NF2 in the liver is intriguing because agrin activates an integrin-FAK-ILK signaling axis to stimulate p21-Activated kinase (PAK1) that subsequently inactivates Merlin [54]. Agrin actively facilitated the interactions of PAK1 and ILK with Merlin, to inactivate the latter’s tumor suppressive abilities [54]. Independent observations revealed that AMOT facilitates the binding of LATS1/2 with Merlin, and the Ser518 phosphorylation prevents it from binding to AMOT, thereby suppressing Merlin’s activity [89]. Interestingly, Merlin is also reported to guide migration and lamellipodium in multicellular organized cells (resembling a growing tumor mass!), suggesting that it responds to collective mechanotransduction [90]. In-vitro data reflects an antagonistic relationship between agrin and Merlin [54]. Possibly, agrin-ILK-PAK1 mechanosignalling pathway serves to counteract the intrinsic mechanotransducing abilities of AMOT:Merlin complex in the context of liver tumor initiation and progression, though more extensive studies are needed to prove the existence of counteractive mechanosignalling. Importantly, ECM crosslinking, integrin clustering, and FAK activation are events that underline tissue stiffness, tumor progression and poor prognosis [91]. Since agrin is localized to activated integrin clusters in liver cancer cells [47], this mechanosignalling axis holds a possible key for nullifying the Hippo pathway to promote YAP-dependent tumorigenesis. 

### 2.5. Agrin Partners with Focal Adhesions to Antagonize the Hippo Pathway

**Figure 4:**
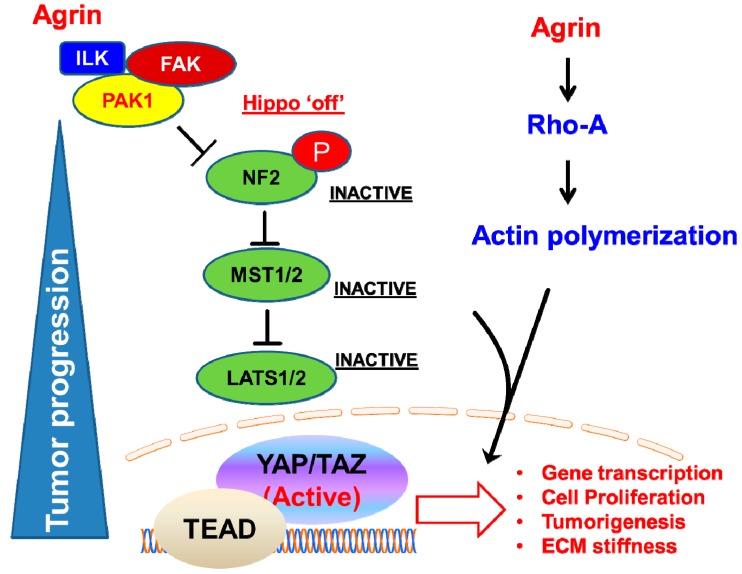
Agrin stimulates integrin-FAK-ILK-PAK1 mechanosignalling pathway to antagonize the Hippo pathway and activate YAP/TAZ mediated gene transcription.

Although integrins-focal adhesions (FA) form the central mechanosensing complex in most cell types, alterations in their molecular composition by mechanotransduction signals and their role in negating the core Hippo components are poorly understood. Agrin, however, may shed new mechanistic insights on how ECM proteins may alter the composition of cell-matrix adhesions and interactions with the Hippo pathway. A working model is that agrin antagonizes the tumor-suppressive functions of Merlin and LATS1/2 kinase by regulating the cell-matrix adhesions [54]. This is attributed by restricting the association of Hippo components within the focal adhesions and activating integrin-FAK-integrin linked kinase (ILK) and p21-activated kinases (PAK1) signaling. Since liver cancer cells actively stabilize FAs shortly after adherence to fibronectin [47], agrin ensures that components of the Hippo pathway are primarily excluded from the activated FAs by engaging ILK-PAK1 [54] (Figure 4). The agrin-induced integrin-ILK-PAK1 axis, in part, inactivated Merlin by promoting an inhibitory phosphorylation at Ser518 [54,92] (Figure 4). In line with this observation, integrin β1 activated Rac-PAK1 pathway has been also shown to inactivate Merlin by inducing a similar phosphorylation in mesenchymal cells [93]. Likewise, diminished Rac1 activity is also observed in agrin-depleted HCC cell lines (data not shown). More excitingly, agrin is also expressed by mesenchymal stem cell (MSC) population [94]. This may indicate that agrin-integrin mechanotransduction loop may inactivate Merlin in many cell/tissue types (Figure 4). Even in the haematopoetic niche, agrin supports the proliferation of MSCs and its deficiency induced apoptosis and impaired haematopoesis [94]. Moreover, agrin clusters the ephrin family of receptor tyrosine kinases (mainly EphB1) to activate integrins that promote adhesion of erythroid cells to macrophages [95], and many of the ephrin receptors have been known to activate YAP [96,97]. Therefore, the associations with integrins and ephrins with agrin will evoke further interests in the field of mechanotransduction, in general.

Mechanoreception and transmission of signalling actively occur at cell junctions involving the acto-myosin complex that also regulates YAP activity, both in flies and mammals [9,35,98,99,100]. However, the role of the Hippo pathway (primarily LATS1/2 kinases) in the regulation of YAP mechanotransduction has been enigmatic. Several reports suggest that YAP’s mechanoresponse is independent of LATS1/2 activity [9,10,51]. This notion primarily arises from the fact that LATS1/2 depletion failed to promote YAP’s nuclear localization in cells cultured on compliant matrices, and YAP mutants resistant to the inhibitory LATS1/2 kinase mediated phosphorylation also show sensitivity to compliant substrates [9,10]. Mechanistically, F-actin mediated cytoskeletal tension was proposed as a parallel “non-canonical” pathway regulating YAP’s localization in response to mechanical changes, independent of the Hippo pathway [9,10]. In contrast, a number of reports suggest that LATS1/2 mediated regulation of actin cytoskeleton is responsible for YAP activity, even under altered mechanical stress [35,100]. F-actin modulation is thought to be dependent on the Hippo pathway in some contexts, such that disruption of F-actin enhanced LATS activity and YAP phosphorylation [35,99,101,102]. In fact, the core Hippo kinases Mst1/2 and LATS1/2 have been shown to bind F-Actin as well [103,104]. In this context, at-least in liver cancer cells, depletion of LATS1/2 diminished the enhanced phosphorylation of YAP at Ser127, suggesting the regulation of YAP phosphorylation by agrin may be partially dependent on the Hippo kinases [54]. It is well known that mechanical tensions across cell junctions regulate the Hippo pathway, YAP localization and activity, in both *Drosophila* and mammals [98,105]; however, the interactions of agrin within these cellular junctions that experience high mechanical strain leading to YAP activity will require further investigations.

In addition, Rho-GTPases act upstream of YAP/TAZ and inhibiting them by *Clostridium botulinium* toxin (C3) abolished YAP/TAZ activity [9]. Rho-GTPases have also been shown to modulate YAP activity downstream to the integrins as well [80,106]. As such, in cells that harbor defective Hippo signaling (such as Merlin null MDA-MB-231), agrin relayed responses to YAP through actin cytoskeletal rearrangements that were dependent on RhoA activity [54] (Figure 4). Presumably, the mechanotransduction pathway of integrins involves actin dynamics in such scenarios. It has become increasingly clear that the integrity of actin-cytoskeleton and RhoA is required for functional YAP/TAZ mechanotransduction [10,11,16,34,35,102,107]. Consistently, recombinant agrin has been reported to induce actin polymerization and fiber formation in the liver cancer cell lines [47,54]. Hence, inhibition of actin-rearrangements and RhoA abolished the agrin- induced YAP mechanoresponsiveness in such situations [54]. As a result, agrin’s mechanotransducing effects on YAP also occurs independent of the Hippo pathway machinery under such contexts. Cumulatively, these studies support a model whereby mechanical signals may be transmitted via a combination of both Hippo pathway dependent/independent fashion and imply that LATS1/2 dependent/independent regulations co-exist dynamically.

### 2.6. YAP as a Potent Downstream Mediator of Agrin’s Functions in Liver Cancer

**Figure 5:**
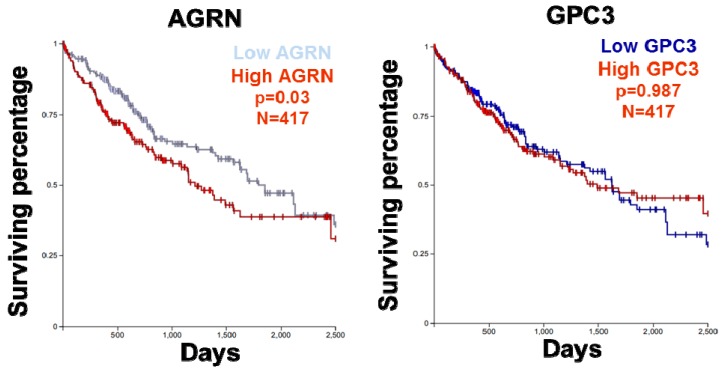
Survival analysis of HCC patients (TCGA datasets) with high or low level of agrin (AGRN) and glypican 3 (GPC3).

**Figure 6:**
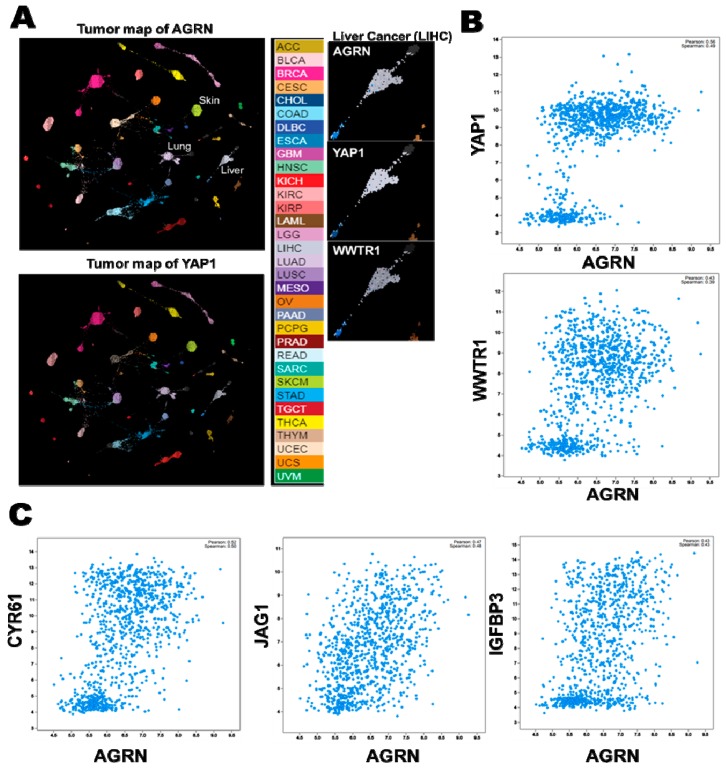
Interactive mapping of Agrin and YAP/TAZ expression profiles across different cancer types. (**A**) Tumor map patterns of AGRN and YAP1 mRNA expression (generated by UCSC tumor map using the TCGA_TARGET_GTEX dataset). The expression patterns of AGRN and YAP1 mRNA are closely matched in several cancer types (Color coded). The panels on the extreme right indicate the relative expression levels of AGRN, YAP1 and WWTR1 (TAZ) in liver cancer (LIHC). The intensity map of expression is indicated below. (**B**) Correlation between AGRN and YAP1/WWTR1 mRNA across cancer cell lines (Cancer Cell Encyclopedia-Novartis/Broad Institute dataset). (**C**) Correlation between agrin and YAP target gene expressions across cancer cell lines.

Several studies have reported the nuclear expression of YAP/TAZ in HCC [108,109,110]. Therefore, as expected from a potent oncogene, YAP functions as a mediator of oncogenic property induced by agrin. Indeed, suppression of YAP activity abolished the majority of oncogenic properties associated with agrin overexpression in liver cancer cell lines. These results suggest that agrin relies on the transcriptional activity of YAP to promote HCC. Clearly, the agrin-induced cellular migratory and invasive capabilities were dependent on YAP activity, as these were significantly reduced in YAP depleted liver cancer cells [47,54]. From a clinical perspective, ECM rigidity is well correlated with malignancy and cancer progression resulting in poor patient outcome [1,91]. Consistently, nuclear YAP and higher agrin expression were observed in the majority of HCC patient tissues that also correlated with a poor prognosis [54,111,112] (Table 1). Compared to other proteoglycans, higher expression of agrin correlated to a poor survival in HCC (Figure 5). More importantly, combined expression of agrin together with YAP target gene(s) accounted for even poorer survival amongst liver cancer patients [54]. Consistently, YAP activated in the stellate cells in response to liver injury promoted hepatic fibrosis, a condition that often precedes liver cancer development [77]. It will be interesting to find out at what stage of cirrhosis and cancer development does agrin expression become prominent? Moreover, the genetic deficiency of Hippo signaling (such as loss of MST1/2) activates YAP to generate a pro-tumorigenic environment that alters macrophage recruitment and immune responses facilitating the development of HCC [113,114,115,116]. Interestingly, agrin depletion enhanced Mst1 phosphorylation in the HCC cell lines [54]. In light of this evidence, the role of agrin in the absence of key Hippo components during the liver cancer development will require extensive future work. Together, enhanced agrin levels and YAP activity may represent key factors that underline stiffened ECM as observed during fibrosis and HCC development. Importantly, agrin is also overexpressed in oral squamous cell carcinoma (OSCC), where YAP/TAZ drives the oncogenic program and confers migratory and invasive potential to OSCC cells [117,118]. Likewise, expression profile analysis using the newly developed tumor map strategy [119] (https://tumormap.ucsc.edu/) indicates that agrin may be expressed in a wide variety of cancers, closely matching the profile of YAP/TAZ (Figure 6A). In fact, robust positive correlation exist between the expression profiles of agrin, YAP/TAZ and several of their targets across different cancer cell lines (Figure 6B,C). Whether agrin and YAP/TAZ team up as oncogenic drivers in other cancer(s) will be one interesting area in cancer research. 

Of potential therapeutic value, targeting agrin through antibody therapy may also inhibit the functions of YAP/TAZ and serve as a potential clinical benefit for HCC treatment in the future. Clearly, proof-of-principle studies have shown that antibodies directed against agrin may have in vivo tumor-inhibitory capabilities [47], although the underlying mechanism of action needs to be defined. The fact that agrin expression is hardly detected in normal human and mouse livers makes it an attractive target for HCC therapy [46,70]. Recently, targeting Glypican-3 (encoded by GPC3), a relative of agrin in the ECM proteoglycan family, that is also frequently overexpressed in HCC has been shown to inactivate YAP, though the exact mechanisms remain unclear [120,121,122,123]. Moreover, given that we are beginning to understand the clinical relevance of elevated agrin expression and mechanisms behind agrin-YAP regulation, targeting agrin through function blocking antibodies holds great promise as additional HCC therapy (Figure 5). However, to understand how generic is agrin secreted and/or expressed in the liver during its tumor initiation stages would be key for effective HCC therapy. 

### 2.7. Influence of Agrin on YAP in other Organs

**Figure 7:**
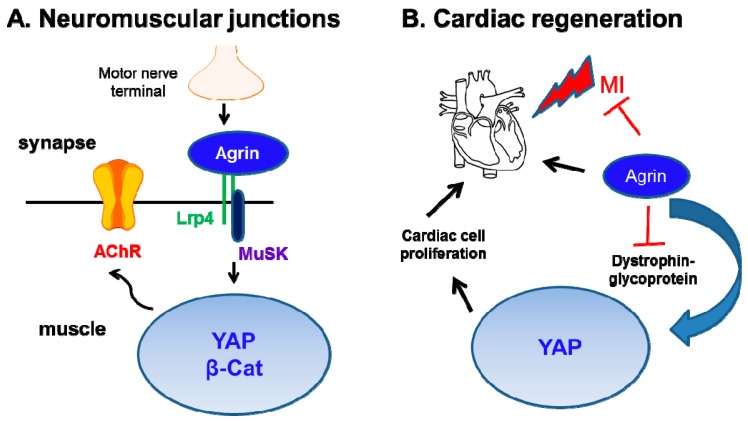
Concerted agrin and YAP activity in the maintenance of NMJs and promoting cardiac regeneration. (**A**) Agrin secretion from motor nerve terminal activates Lrp4-MuSK signaling. This potentiates YAP and possibly β-catenin activity in the nucleus of muscle cells, thereby, aggregating Acetylcholine receptors (AchR) and propagating nerve impulse to the muscle cells. (**B**) Though adult hearts have minimal agrin expression compared to that during neonatal stages, stimulation with recombinant agrin has protective values against myocardial infarction (MI) in adult mice hearts. Mechanistically, agrin binds to and inhibits dystroglycan complex, thereby, shifting YAP into the nucleus to engage cardiomyocyte proliferation and regeneration.

In addition to cancer models, the role of agrin as a critical regulator of YAP function has been concomitantly discovered in multiple organs. Firstly, a genetic deletion of YAP in muscle cells caused severe defects in the development of NMJs [124]. The pre-synaptic deficits and loss of AChR clustering in YAP mutant mice closely resembled that of agrin-deficient mice [39], thereby supporting a functional link between agrin and YAP as its downstream mediator of functions in NMJ development. Moreover, muscle YAP was shown to orchestrate post-synaptic differentiation and regeneration of NMJs followed by a nerve injury. Lack of YAP also reduced β-catenin activity in the NMJs. On a broader perspective, YAP and β-catenin not only orchestrate the NMJ development but also serve as potent drivers of liver cancer (Figure 7). As such, activating mutations in β-catenin are detected in 21% of HCC cases [125].

Therefore, it is tempting to speculate that agrin may control NMJ and cancer progression through the concerted activities of YAP and β-catenin. Whether agrin regulates β-catenin activity needs further experimentation. Whether agrin-YAP loop senses the stiffness of NMJs required for efficient impulse transmission is speculative at this stage. Furthermore, whether agrin stimulates YAP activity for regeneration of NMJs following nerve injury will be of potential future interests.

Secondly, Bassat et al., identified an exciting role of agrin in promoting regeneration post-myocardial infarction (MI) in cardiomyocytes [126]. In a related study, dystrophin-glycoprotein complex (consisting of α- and β-dystroglycans receptors for agrin [70,127]), has been shown as a part of the Hippo pathway to inhibit YAP activity, and thereby restricting the proliferation of cardiomyocytes [128]. Mechanistically, high levels of agrin during neonatal stages of heart development not only inhibits the dystrophin glycoprotein complex to release YAP from the inhibitory effects of the dystroglycan complex, therefore, potentiating YAP’s nuclear localization in cardiac cells to promote regenerative capacity. This agrin-induced YAP activity stimulated proliferation and dedifferentiation of the cardiomyocytes [126]. Consistent with its role in liver cancer, soluble agrin also potentiated nuclear YAP localization in cardiac cells, and significantly rescued damage incurred from experimental MI [126] (Figure 7). However, it needs to be explored whether dystrophin complex forms a part of Hippo tumor suppressor pathway in liver or other organs. More importantly, these studies potentially create avenues for an exciting research platform that may include: (i) whether agrin nullifies the dystrophin-glycoprotein complex in a similar fashion to promote tumorigenesis and invasiveness; (ii) whether agrin enhances the stiffness of cardiac cells and basal lamina surrounding muscle cells; and an existence of a temporal stiffness pattern dependent on the age of the cardiac cells also remains unclear; (iii) do focal adhesions promote agrin-induced cardiac regeneration? The loss of agrin in adult cardiomyocytes may represent part of the basis for the limited regenerative capabilities of mature heart muscles. Therefore, strategies aimed at the localized enrichment of agrin within cardiac tissues to re-activate YAP to restore heart failure will be of immense clinical value. Presently, these findings support that agrin is a link between extracellular matrix and YAP activation during developmental stages in multiple organs. 

Agrin also represents a key proteoglycan in the glomerular basement membrane (GBM) in kidneys where it combines with integrins, laminins and nidogen [70,129]. In addition, conditional knock-out of YAP in mouse kidneys has pronounced nephrogenic abnormalities that phenocopies that of knockout of Cdc42, a small GTPase downstream to RhoA [130]. Interestingly, agrin may cooperate with Arp2/3 and Cdc42 in the liver cancer cell lines to drive their migratory potential [47]. Moreover, deficiency of Merlin and LATS1/2 also promote an unusual branching morphology in kidneys that are rescued upon YAP overexpression [131]. Given that agrin profoundly regulates Cdc42 and Merlin to activate YAP as a mechanotransduction pathway [47,54,130], it would be very interesting to study the role of agrin-YAP mechanotransduction pathway in kidney development and tumorigenesis.

## 3. Conclusions

The precise role of secreted ECM proteins in engaging mechanical signals in cancer, NMJs and cardiomyocytes is poorly characterized. Irrespectively, these recent findings evoke new insights on the role of secreted agrin in sustaining the nuclear levels of YAP under mechanically stressed conditions such as in cancer, nerve impulse propagation, and organ development and regeneration. These findings advance our understanding on the role(s) of soluble ECM factors (particularly agrin) that facilitate ECM stiffness, activate key developmental signaling or oncogenes, and thereby promote concerted signaling and proliferation. Understanding the agrin-YAP mechanotransduction loop may help to develop therapeutic strategies against diseases including liver cancer. More importantly, extensive future research is required to understand the influence of cell mechanics involving agrin and YAP activities in other organs and cancer types.

## Figures and Tables

**Table 1:** Distinction between normal and HCC liver tissue matrices.

Normal Liver Tissue	Hepatocellular Carcinoma (HCC)
No distinct basement membrane, low Agrin levels, less collagen crosslinking, cytoplasmic YAP/TAZ, low contractile forces, compliant ECM	High Agrin levels and fibrillary collagen content, nuclear YAP/TAZ, mechanosignalling activated, higher contractile forces, stiffened ECM
